# Association between physical activity level and cardiovascular risk factors in adolescents living with type 1 diabetes mellitus: a cross-sectional study

**DOI:** 10.1186/s12933-021-01255-0

**Published:** 2021-03-12

**Authors:** Nana Wu, Shannon S. D. Bredin, Veronica K. Jamnik, Michael S. Koehle, Yanfei Guan, Erin M. Shellington, Yongfeng Li, Jun Li, Darren E. R. Warburton

**Affiliations:** 1 Physical Activity Promotion and Chronic Disease Prevention Unit, The University of British Columbia, Vancouver, BC Canada; 2 School of Kinesiology and Health Science, York University, Toronto, ON Canada; 3 School of Kinesiology, The University of British Columbia, Vancouver, BC Canada; 4 College of Sports and Health, Shandong Sport University, Ji’nan, Shandong China; 5 School of Sport Social Science, Shandong Sport University, Ji’nan, Shandong China

**Keywords:** Physical activity, Cardiovascular health, Type 1 diabetes mellitus

## Abstract

**Background:**

Type 1 diabetes mellitus (T1DM) is associated with an increased risk for cardiovascular disease (CVD) related morbidity and premature mortality. Regular physical activity plays an important role in the primary and secondary prevention of CVD, improving overall health and wellbeing. Previous observational studies have examined the associations between self-reported physical activity and CVD risk factors in largely adult Caucasian populations. However, limited work has evaluated the relationship between objectively measured physical activity and CVD risk factors in other ethnicities, particularly Chinese youth living with T1DM.

**Methods:**

This cross-sectional study assessed CVD risk factors, physical activity, and aerobic fitness (and their associations) in Chinese youth living with T1DM (n = 48) and peers (n = 19) without T1DM. Primary outcomes included blood pressure, lipid profiles, and physical activity (accelerometry). Statistical differences between groups were determined with chi-square, independent-samples t-tests, or analysis of covariance. The associations between aerobic fitness, daily physical activity variables, and CVD risk factors were assessed with univariate and multivariate linear regression analyses.

**Results:**

Results were summarized using means and standard deviation (SD) for normally distributed variables and medians and 25–75th quartile for non-normally distributed variables. In comparison to peers without diabetes, youth living with T1DM showed higher levels of total cholesterol (3.14 ± 0.67 vs. 4.03 ± 0.81 mmol·L^-1^, *p* = 0.001), low-density lipoprotein cholesterol (1.74 ± 0.38 vs. 2.31 ± 0.72 mmol·L^-1^, *p* = 0.005), and triglycerides (0.60 ± 0.40 vs. 0.89 ± 0.31 mmol·L^-1^ *p* = 0.012), and lower maximal oxygen power (44.43 ± 8.29 vs. 35.48 ± 8.72 mL·kg^-1^·min^-1^, *p* = 0.003), total physical activity counts (451.01 ± 133.52 vs. 346.87 ± 101.97 counts·min^-1^, *p* = 0.004), metabolic equivalents (METs) (2.41 ± 0.60 vs. 2.09 ± 0.41 METs, *p* = 0.033), moderate-to-vigorous intensity physical activity [MVPA: 89.57 (61.00–124.14) vs (53.19 (35.68–63.16) min, *p* = 0.001], and the percentage of time spent in MVPA [11.91 (7.74–16.22) vs 8.56 (6.18–10.12) %, *p* = 0.038]. The level of high-density lipoprotein cholesterol was positively associated with METs (β = 0.29, *p* = 0.030, model R^2^ = 0.168), and the level of triglycerides was negatively associated with physical activity counts (β = − 0.001, *p* = 0.018, model R^2^ = 0.205) and METs (β = − 0.359, *p* = 0.015, model R^2^ = 0.208), and positively associated with time spent in sedentary behaviour (β = 0.002, *p* = 0.041, model R^2^ = 0.156) in persons living with T1DM.

**Conclusions:**

Chinese youth with T1DM, despite their young age and short duration of diabetes, present early signs of CVD risk, as well as low physical activity levels and cardiorespiratory fitness compared to apparently healthy peers without diabetes. Regular physical activity is associated with a beneficial cardiovascular profile in T1DM, including improvements in lipid profile. Thus, physical activity participation should be widely promoted in youth living with T1DM.

## Background

Type 1 diabetes mellitus (T1DM) is associated with high risk of microvascular and macrovascular complications, as well as other cardiovascular disease (CVD) risk factors, including obesity, hypertension, hyperglycemia, dyslipidemia, insulin resistance, and physical inactivity [1]. Cardiovascular disease is the most frequent cause of premature death and disability in this population [2]. In fact, the childhood-onset of T1DM has been associated with a higher risk of developing CVD in adulthood [3]. In a cross-sectional study, 76% of children and adolescents with T1DM were found to have one or more risk factors for CVD (i.e., obesity, hypertension, hyperglycemia, or dyslipidemia) [4]. Therefore, there is an urgent need for prevention and treatment strategies to reduce CVD risk factors in children and youth living with T1DM.

One recent systematic review and meta-analysis showed that exercise training can decrease risk factors for CVD by improving cardiovascular fitness, endothelial function, and vascular health in youth living with T1DM [1]. Furthermore, the review also demonstrated that exercise training can reduce the severity of CVD risk factors (such as obesity and body composition, high blood pressure, worsened lipid lipoprotein profile, and systemic inflammation) [1]. The American College of Sports Medicine guidelines for exercise testing and prescription recommend that individuals living with T1DM undertake 150 minutes of exercise at 40–59% of their oxygen uptake reserve (VO_2_R) or 75 minutes of vigorous intensity exercise (60–89%VO_2_R) per week, or 30 minutes or more of daily low to moderate intensity physical activity participation [5]. Moreover, the 2018 Diabetes Canada clinical practice guidelines recommend that even smaller amounts of physical activity can provide some health benefits [6].

Despite the clear potential health benefits, individuals living with T1DM may fear or be discouraged from regular physical activity participation due to the lack of adequate knowledge about exercise management and concerns of hypoglycemic episodes [7]. According to a large German/Austrian uncontrolled study of self-reported physical activity, 82.7% of youth aged 3–18 years with T1DM did not meet daily physical activity recommendations and regular physical activity was linked with a beneficial CVD risk profile [8]. In a Canadian study, adolescents with T1DM, surveyed using the Habitual Activity Estimation Scale, were shown to spend more time being less active than their peers without T1DM and physical activity was associated with improved CVD risk profile [9]. Firstly, objective assessments of physical activity such as continuous heart rate monitoring and triaxial accelerometry (activity monitors that measure position and motion) provide more valid data than self-reports of physical activity [10]. Secondly, CVD risk factors may vary between ethnic groups. One recent study found that African immigrants had lower age-standardized hypertension, diabetes mellitus, overweight/obesity, high cholesterol, and current smoking prevalence than African Americans [11]. Collectively, there is limited research examining the relationships between objectively measured physical activity and CVD risk profile, particularly among Chinese youth living with T1DM.

Accordingly, the primary objective of this study was to assess CVD risk factors in Chinese youth living with T1DM in comparison with apparently healthy peers not living with T1DM. The second objective was to evaluate the relationship between objectively measured daily physical activity levels and markers of CVD. We hypothesized that the CVD risk profile in Chinese youth with T1DM would be proatherogenic compared with the profile of apparently healthy peers without diabetes, and that higher physical activity levels would be associated with improved CVD risk factors among Chinese youth with T1DM.

## Methods

### Study design and participants

We conducted a cross-sectional study including 48 adolescents living with T1DM (World Health Organization (WHO) criteria) and 19 apparently healthy peers without diabetes (aged 12 to 17 years). The participants with T1DM were all Chinese recruited via referral, snowball sampling, and social media. Individuals living with T1DM for at least 6 months with glycosylated hemoglobin (HbA1c) greater than or equal to 7.5% in the last three months, with normal renal function, and free from previous CVD and chronic kidney disease were eligible for participation. Participants with significant diabetic complications (diabetic foot, retinopathy, severe neuropathy), uncontrolled hypertension, diabetic keto-acidosis, CVD (defined as any form of clinical coronary heart disease, stroke or peripheral vascular disease), severe hypoglycemia episodes within the past 3 months were excluded. Participants on lipid lowering therapy were also excluded.

Peers without diabetes (apparently healthy) participants were recruited from local schools and via snowball sampling and were selected to match the diabetic group in age and sex distribution. This apparently healthy group had no known history of chronic disease and no clinical or laboratory evidence of hypertension, cardiac disease, or other problems that would have contraindicated or limited their participation in regular physical activity. Participants that took any medications, which could influence cardiovascular function, lipid lipoprotein profiles, and/or glucose metabolism were excluded from the study.

Recruitment of adolescents with T1DM was done via advertisements in social media platform (e.g., Diabetes Wechat groups), by distributing initial letters to the individuals with T1DM who visited local hospitals, and via snowball sampling. A total of 88 individuals with T1DM aged 12–17 years were recruited for this study, and nine of whom had one or more exclusion criteria were excluded. Seventy-nine participants were eligible for participation and 48 consented to participate the study. In total, 67 participants were recruited, of which 48 had T1DM and 19 participants were peers without diabetes.

### Anthropometric measurements

Height was measured in bare feet to the nearest 0.1 cm using a wall-mounted stadiometer. Body mass index (BMI) was calculated as weight (kg) divided by height squared (m^2^) and adjusted for age and sex to give a BMI standard deviation score (BMI z-score). BMI z-score was calculated using the WHO Reference 2007 growth data for the age group 5–19 years with SPSS software (https://www.who.int/growthref/tools/en/). Waist circumference was measured in triplicate with a flexible tape at the midpoint between the lower margin of the last palpable rib and the top of the iliac crest following WHO guidance [12]. Blood pressure was measured after 10 min of rest in the seated position and the average of three measurements taken 1 min apart was used in the analysis. The cuff was chosen to be of the appropriate size for the participant’s upper arm, with a bladder width that is at least 40% of the arm circumference at a point midway between the olecranon and the acromion and a bladder length to cover 80–100% of the circumference of the arm.

### Body composition assessment

Body composition measurements were performed by dual energy X-ray absorptiometry (DXA) using the iDXA instrument (GE Medical Systems, Madison, WI) with Encore 2011 software (version 13.6). All study participants underwent a whole-body scan (Lunar iDXA, GE Healthcare, WI), in which the participant was placed in the supine position, centralized in the DXA scan table.

### Puberty and diabetes assessment

Puberty was assessed via a validated self-report questionnaire using pubertal staging images and the children were categorized as prepubertal (Tanner 1), in early puberty (Tanner 2), mid puberty (Tanner 3–4) or post puberty (Tanner 5) [13]. This questionnaire has been validated in Chinese children [14]. Participants were asked to complete a questionnaire upon arrival at the testing location, which included: diabetes history, age, duration of diabetes, complications, insulin regimen, medications, and physical activity levels.

### Biochemical investigations

All participants were studied after a 12-h overnight fast and, in the case of T1DM individuals, before their morning insulin injections. Fasting capillary (fingertip) blood glucose samples were taken for analysis of HbA1c, total cholesterol, LDL-C, HDL-C, and triglycerides. Total cholesterol, LDL-C, HDL-C and triglycerides were analyzed using the Cardiochek PA Blood Analyser (Polymer Technology Systems Inc., Indianapolis, IN, USA) and HbA1c were analyzed using the A1cNow+ (Metrika Inc., Sunnyvale, CA, USA). Both devices have been validated previously [15, 16].

### Physical activity assessment

Physical activity levels were objectively measured (24 h per day) using triaxial accelerometers (wGT3x-BT ActiGraph LLC, Pensacola, FL, USA). Participants were asked to wear the accelerometers on their non-dominant wrist for seven consecutive days (5 weekdays), except in the water, as the device is water-resistant, but not waterproof. We collected data at 50 Hz, as this sampling frequency has shown to sufficiently capture body movement and allow for five weekdays and two weekend days of data collection [17]. Participants were given a paper calendar-style tracking log on which they were instructed to write down the time they put the accelerometer on and the time they removed it in order to support wear-time compliance.

Data were downloaded with the manufacturer’s software (ActiLife Version 6) and processed using 60-s epochs to derive the following daily physical activity parameters: metabolic equivalent of task (METs, kcal·h^−1^·kg^−1^; as an index of the intensity of activities), total daily physical activity counts (CPM), the percentage of time spent in sedentary behaviour, daily average time spent in light intensity physical activity, daily average time spent in moderate-to-vigorous intensity physical activity (MVPA), and the percentage of time spent in MVPA. Sedentary behaviour period (≤ 100 CPM), light intensity physical activity (101–2295 CPM), moderate intensity physical activity (2296–4011 CPM) and vigorous intensity physical activity (≥ 4012 CPM) established cut-offs were used [18]. Moderate-to-vigorous intensity physical activity time accumulated in bouts (prolonged periods) of 10 or more consecutive minutes following physical activity guidelines was also derived [19]. Sedentary time accumulated in bouts of 20 or more consecutive minutes, which has been shown to have a negative effect on cardio-metabolic biomarkers [20], was also derived. The non-wear period was defined as a minimum of 60 min of continuous zero counts according to ActiLife’s default option and at least 600 min of wear time per day without excessive counts (> 20,000 CPM) was required to be considered valid [21]. At least three valid wear days were required to be included in the analysis.

Resting energy expenditure was estimated from accelerometer counts and age-specific prediction equations to derive the metabolic equivalent of MET intensity levels. The equation was: METs = 2.757 + (0.0015*CPM)—(0.08957*age)—(0.000038*CPM*age) [22].

### Physical fitness assessment

Participants were screened for any cardiovascular complications and the readiness for exercise testing using the Physical Activity Readiness Questionnaire for Everyone (PAR-Q+) [23] prior to physical fitness test. The Leger 20-metre shuttle run test was used for aerobic fitness assessment. The frequency of the sound signals increased in such a way that running speed was increased by 0.5 km·h^-1^ each minute from a starting speed of 8.5 km·h^-1^. The test stopped when the participants were no longer able to follow the set pace. If patients experienced hypoglycemia during their laboratory stay, a 15 g carbohydrate bolus was administered (Glucose drinks, Henan Three Connaught food Co., LTD, China). Hypoglycemia was defined as a blood glucose concentration of ≤ 3.9 mmol·L^-1^ and hyperglycemia ≥ 10.9 mmol·L^-1^ [24].

### Statistical analysis

All statistical analyses were performed using the SPSS 20 for Windows (Statistical Package for the Social Sciences). Data were screened for normal distribution. The chi-square test was used for comparison of proportion (categorical variable including sex and pubertal status) between groups, and independent-samples t-tests were used for comparison of the continuous variables (i.e., age, height, body composition, BMI, cardiovascular risk factors and daily physical activity variables) between groups. Cohen’s d effect size was calculated for t-tests and described as small (d = 0.2), moderate (d = 0.5) and large (d = 0.8) based on benchmarks suggested by Cohen [25]. We included BMI z-score as a covariate in an analysis of covariance (ANCOVA) for statistical differences between groups by reducing the error variance because BMI z-score correlates with the blood pressure, daily physical activity variables, lipid profiles, and aerobic fitness. The non-parametric test (Mann-Whitney) was used to compare time spent in MVPA and the percentage of time in MVPA among groups as the distributions were not normal. Effect size statistic for the Mann–Whitney test is r, which was calculated by dividing Z by the square root of total number of samples (r = Z/√N) and described as small (r = 0.1), moderate (r = 0.3) and large (r = 0.5). Results were summarized using means and standard deviation (SD) for normally distributed variables, medians and 25–75th quartile for non-normally distributed variables and using frequencies and percentages for categorical variables.

The associations between aerobic fitness, daily physical activity variables and cardiovascular risk factors were assessed with univariate linear regression analysis (Step 1) in T1DM group and apparently healthy peers without diabetes, and multivariate linear regression analysis (Step 2) adjusting for age, sex, insulin dose, BMI z-score and pubertal stage in individuals with T1DM and adjusting for age, sex, BMI z-score and pubertal stage in apparently healthy peers not living with diabetes. The probability was considered to be statistically significant at *p* value < 0.05.

## Results

The demographic characteristics and laboratory results of individuals with T1DM and apparently healthy peers without diabetes are presented in Table 1. Specific descriptive data in youth with T1DM are presented in Table 2. No significant differences were shown between groups for age, sex, pubertal stage, height, body mass, body fat percentage, BMI, waist circumference, and blood pressure (all *p* > 0.05). T1DM participants showed significantly higher values of total cholesterol (4.03 ± 0.81 vs. 3.14 ± 0.67 mmol·L^−1^, *p* = 0.001, d = 1.20), LDL-C (2.31 ± 0.72 vs. 1.74 ± 0.38 mmol·L^−1^, *p* = 0.005, d = 0.99), triglycerides (0.89 ± 0.31 vs. 0.60 ± 0.40 mmol·L^−1^, *p* = 0.012, d = 0.81) compared to apparently healthy peers without diabetes.

**Table 1:** Comparison of descriptive data between young people with type 1 diabetes mellitus and apparently healthy peers without diabetes

	T1DM (n = 48)		Peers without diabetes (n = 19)			
	Mean	SD	Mean	SD	*p*	Adjusted *p*
Sex (male/ female) (%)	37.5/62.5		42.1/57.9		0.129	
Pubertal stage (n)					0.543	
Stage 1 (Not started)	18		7			
Stage 2 (Barely started)	8		1			
Stage 3 (Definitely underway)	12		6			
Stage 4 (Seems completed)	5		1			
Stage 5 (Completed)	5		4			
Ages (y)	14.02	2.89	13.58	3.46	0.601	
Body mass (kg)	49.48	12.56	52.34	15.45	0.452	
Height (m)	1.60	0.13	1.59	0.13	0.772	
BMI (kg∙m^−2^)	18.97	3.10	20.38	3.32	0.119	
BMI z-score	− 0.27	1.16	0.50	1.01	0.018^*^	
Body fat (%)	29.28	9.54	28.42	6.61	0.765	0.084
Waist circumference (cm)	67.63	7.96	73.65	8.22	0.055	0.151
Systolic blood pressure (mmHg)	106.07	16.72	107.07	15.45	0.837	0.778
Diastolic blood pressure (mmHg)	65.24	11.65	65.75	9.88	0.878	0.821
Total cholesterol (mmol∙L^−1^)	4.03	0.81	3.14	0.67	0.001^*^	0.001
HDL-C (mmol∙L^−1^)	1.48	0.28	1.29	0.42	0.078	0.184
LDL-C (mmol∙L^−1^)	2.31	0.72	1.74	0.38	0.005^*^	0.008
Triglycerides (mmol∙L^−1^)	0.89	0.31	0.60	0.40	0.012^*^	0.007
Estimated VO_2_max (mL∙kg^−1^∙min^−1^)	35.48	8.72	44.43	8.29	0.003^*^	0.003
METs (kcal∙h^−1^∙kg^−1^)‡	2.09	0.41	2.41	0.60	0.066	0.075
Physical activity counts (CPM)‡	346.87	101.97	451.01	133.52	0.004^*^	0.033
Light physical activity (min∙day^−1^)‡	335.93	120.16	449.33	89.55	0.002^*^	0.003
Percentage of time spent in Sedentary behaviour (%)‡	36.34	11.04	28.86	12.56	0.04^*^	0.095

Data are presented as means and standard deviation (SD)

*T1DM* type 1 diabetes mellitus, *n* number, *y* year, *BMI* body mass index, *HDL-C* high-density lipoprotein cholesterol, *LDL-C* low-density lipoprotein cholesterol, *VO*_*2*_*max* maximal aerobic power, *METs* metabolic equivalents, *CPM* counts-per-minute, *adjusted p* ANCOVA adjusted for BMI z-score

^*^Statistically significant difference (*p* < 0.05) between T1DM and apparently healthy peers without diabetes

‡Sixteen of 19 apparently healthy participants not living with diabetes and 40 of 48 participants with T1DM had valid physical activity data

**Table 2:** Specific descriptive data in young people living with type 1 diabetes mellitus

	Type 1 diabetes mellitus (n = 48)	
	Mean	SD
Diabetes duration (years)	3.64	2.29
Pubertal Stage (n)		
Prepubertal (Tanner 1)	18	
Early puberty (Tanner 2)	8	
Mid-puberty (Tanner 3‐4)	17	
Post-puberty (Tanner 5)	5	
MDI	30	
CSII	18	
CGM	26	
SMBG	22	
HbA1c (mmol∙mol^−1^)	61	9
HbA1c (%)	7.70	2.47
Insulin dose (unit∙kg^−1^∙day^−1^)	0.87	0.29

Data are presented as means and standard deviation (SD)

*MDI* multiple daily injection, *CSII* continuous subcutaneous insulin infusion, *CGM* Continuous Glucose Monitoring, *SMBG* self-monitoring of blood glucose, *HbA1c* Glycosylated hemoglobin

Individuals living with T1DM had significantly lower maximal aerobic power (VO_2_max) (35.48 ± 8.72 vs. 44.43 ± 8.29 mL·kg^−1^·min^−1^, *p* = 0.003, d = 1.05), total daily physical activity counts (346.87 ± 101.97 vs. 451.01 ± 133.52 CPM, *p* = 0.004, d = 0.88), and daily light physical activity (335.93 ± 120.16 vs. 449.33 ± 89.55 min, *p* = 0.002, d = 1.07), with higher percentage of time spent in sedentary behaviour (36.34 ± 11.04 vs. 28.86 ± 12.56, *p* = 0.04, d = 0.63) compared with healthy participants. In persons living with T1DM, median MVPA [(53.19 (35.68—63.16) vs 89.57 (61.00—124.14) min, *p* = 0.001, r = 0.40] and median percentage of time spent in MVPA [(8.56 (6.18–10.12) vs 11.91 (7.74—16.22) %, *p* = 0.038, r = 0.25] were significantly lower compared to apparently healthy peers without diabetes. Because of the observed differences in BMI z-score, the *p* value of main outcomes adjusted for the differences were also calculated (Table 1). ANCOVA of controlling BMI z-score did not impact the overall significance of the main outcomes between two groups (Table 1).

We investigated the within-group relationships between CVD risk factors (blood pressure, HbA1c, HDL-C, LDL-C, total cholesterol, and triglycerides) and aerobic fitness or daily physical activity variables with bivariate analysis. For participants with T1DM, the METs correlated positively with HDL-C (r = 0.410, *p* = 0.030) and negatively with triglycerides (r = − 0.456, *p* = 0.015) (Table 3). Also, there is a significant correlation between triglycerides and time spent in sedentary behaviour (r = 0.395, *p* = 0.041), and physical activity counts (r = − 0.453, *p* = 0.018) (Table 3). No similar relationships were shown in healthy participants (*p* > 0.05).

**Table 3:** Correlation between cardiovascular risk factors and daily physical activity variables in young people living with type 1 diabetes mellitus

		(1)	(2)	(3)	(4)	(5)
(1) HDL-C	Pearson’s r	–				
	p	–				
(2) Triglycerides	Pearson’s r	− 0.450	–			
	p	0.010^*^	–			
(3) METs	Pearson’s r	0.410	− 0.456	–		
	p	0.030^*^	0.015^*^	–		
(4) Physical activity counts	Pearson’s r	0.331	− 0.453	0.837	–	
	p	0.091	0.018^*^	0.000^*^	–	
(5) Time spent in sedentary behavior	Pearson’s r	− 0.254	0.395	− 0.509	− 0.446	–
	*p*	0.201	0.041^*^	0.003^*^	0.010^*^	–

*HDL-C* high-density lipoprotein cholesterol, *METs* metabolic equivalents

^*^Statistically significant difference (*p* < 0.05)

Linear regression models are presented in Table 4 and showed that HDL-C in persons with T1DM were positively associated with METs (β = 0.29, *p* = 0.030, model R^2^ = 0.168), but the results changed after adjusting for age, sex, pubertal stage, BMI z-score (β = 0.305, *p* = 0.195, model R^2^ = 0.305). Linear regression models adjusted for age, sex, pubertal stage, BMI z-score, and insulin treatment showed that there was a trend for a negative association between HDL-C in T1DM and time spent in sedentary behaviour (β = − 0.002, *p* = 0.060, model R^2^ = 0.597). HDL-C in apparently healthy peers without diabetes was not significantly associated with daily physical activity variables and VO_2_max. Triglycerides were negatively associated with daily physical activity counts (β = − 0.001, *p* = 0.018, model R^2^ = 0.205) and METs (β = − 0.359, *p* = 0.015, model R^2^ = 0.208), positively associated with time spent in sedentary behaviour (β = 0.002, *p* = 0.041, model R^2^ = 0.156) in persons with T1DM, but the results changed to non-significance after adjusting for age, sex, pubertal stage, BMI z-score (Table 5).

**Table 4:** Linear regression model examining the association between HDL-C and physical activity variables after adjusting by potential confounders in young people living with type 1 diabetes mellitus

Independent variables	Dependent variable: HDL-C					
	β	95%CI		*p*	Adjusted R^2^	R^2^
METs (kcal∙h^−1^∙kg^−1^)						
Step 1 (unadjusted)	0.290	0.030	0.551	0.030	0.136	0.168
Step 2 (adjusted^†^)	0.305	− 0.172	0.781	0.195	0.537	0.305
Sedentary behaviour (min∙day^−1^)						
Step 1 (unadjusted)	− 0.001	− 0.003	0.001	0.201	0.027	0.065
Step 2 (adjusted^†^)	− 0.002	− 0.003	0.000	0.060	0.383	0.597

*METs* metabolic equivalents, *HDL-C* high-density lipoprotein cholesterol, *β* estimated value, *CI* confidence interval, *R*^*2*^ coefficient of determinations

^†^adjusted for age, sex, insulin dose, BMI z-score, and pubertal stage

**Table 5:** Linear regression model examining the association between triglycerides and physical activity variables after adjusting by potential confounders in young people living with type 1 diabetes mellitus

Independent variables	Dependent variable: Triglycerides					
	β	95%CI		*p*	Adjusted R^2^	R^2^
Physical activity counts (CPM)						
Step 1 (unadjusted)	− 0.001	− 0.003	0.000	0.018	0.173	0.205
Step 2 (adjusted ^†^)	− 0.001	− 0.002	0.001	0.344	0.178	0.463
METs (kcal∙h^−1^∙kg^−1^)						
Step 1 (unadjusted)	− 0.359	− 0.641	− 0.077	0.015	0.178	0.208
Step 2 (adjusted^†^)	− 0.272	− 0.839	0.295	0.326	0.202	0.468
Sedentary behaviour (min∙day^−1^)						
Step 1 (unadjusted)	0.002	0.000	0.003	0.041	0.122	0.156
Step 2 (adjusted^†^)	0.001	− 0.001	0.003	0.299	0.187	0.468

*CPM* counts-per-minute, *METs* metabolic equivalents, *β* estimated value, *CI* confidence interval, *R*^*2*^ coefficient of determinations

^†^Adjusted for age, sex, insulin dose, BMI z-score, and pubertal stage

## Discussion

Similar to the finding in other ethnicities [8, 9], increased levels of risk factors for CVD were found in Chinese youth living with T1DM compared with peers without diabetes. A significant correlation among physical activity levels and components of lipid profile was found when confounders of age, sex, pubertal stage, BMI z-score, and insulin treatment were included. Our findings emphasize the importance of promoting daily physical activity among Chinese youth living with T1DM.

A large proportion (45%) of T1DM patients are exposed to a high risk of early all-cause mortality and premature cardiovascular disease [26, 27]. Adults living with T1DM have a high prevalence of dyslipidemia [28, 29]. Youth with T1DM have proteomic alterations in their HDL compared peers without diabetes and are at increased risk of CVD [30]. Corroborating this, our results showed that Chinese youth living with T1DM have increased total cholesterol, LDL-C, and triglycerides compared to apparently healthy peers without diabetes. Similarly, a previous study showed that Caucasian youth with T1DM (aged 5–15 years) had elevated values of total cholesterol and LDL-C compared to peers without diabetes [31]. Lipids levels are very important in predicting adverse cardiovascular outcomes [32]. Previous studies showed that LDL-C independently correlated with abnormal plethysmography responses [33], endothelial dysfunction [34], carotid intimal medial thickness [35], and aortic intimal medial thickness [36] in youth living with T1DM. Moreover, Katz et al. showed lack of knowledge and parental concerns regarding controlling blood pressure and lipid levels at a young age as patient-related reasons for undertreatment of CVD risk factors in youth with T1DM [37]. Of note, the median duration of diabetes in our study was less than 4 years, which suggests that the functional changes in lipid profiles start very early in the course of the disease and likely deteriorate to over time, previous studies have similarly demonstrated atherogenic lipoprotein profiles in youth with a short duration of diabetes [29], which highlights the importance of optimizing the management of T1DM for lifelong habits and increases awareness of early treatment of CVD risk factors in youth with T1DM.

The management of T1DM is challenging as individuals with T1DM must strategically manage their insulin administration, carbohydrate intake, ketones, exercise, and possible other hormones to maintain their blood glucose levels in the target range [38]. Regular exercise is an important and integral component of effective treatment of T1DM, but glycemic management of exercise is particularly difficult for individuals with T1DM. Because determinants of the direction (increase/decrease) and magnitude of the glycemic response to exercise are variable and based on several factors, including the duration and intensity (high vs. moderate or low intensity) of exercise, individual’s characteristics (endogenous insulin sensitivity, and the effect of aerobic fitness to increase insulin sensitivity), and contextual factors (pre-exercise blood glucose level, insulin- and carbohydrates-on-board, and concentration of counter regulatory hormones) [39]. Consequently, the role of exercise in glycemic control is less clearly defined. Our results showed no significant associations between daily physical activity variables and HbA1c in youth with T1DM. Similar results have been demonstrated in previous studies. Ligtenberg et al. found that glycemic control was not associated with physical activity in adults (aged 18–45 years) living with T1DM [40]. Wieliczko et al. reported that there was no correlation between the time spent every week participating in sports and glycated hemoglobin levels in children and adolescents with T1DM [41]. On the contrary, Herbst et al. found increasing physical activity was associated with better HbA1c levels in 23,251 children living with T1DM [8]. Valerio et al. have reported that regular physical activity was associated with better metabolic control and lipid profile in 138 children and adolescents with T1DM [42]. One of the possible reasons for the inconsistency could be differences in sample size between studies, with larger sample sizes being required to find significant findings. The other reasons could be possible variation in carbohydrate intake and insulin dosage (for avoiding hypoglycaemia episodes that caused by exercise), stress or stricter medical monitoring, which may all influence glycaemic status.

Regular exercise, physical activity participation, and reduced sedentary behaviour are important for CVD risk management [43]. Physical activity can improve the metabolic profile, bone mineral density, cardiorespiratory fitness and insulin sensitivity while lowering mortality risk in children with T1DM and physical activity habits developed during childhood and the associated health benefits may carry forward into adulthood [44]. Contrary to this belief, a recent study with small sample size (n = 14) indicates that vigorous exercise (56% of VO_2_max) in hot environment (35 ℃, relative humidity ~ 20%) can exacerbate reductions in cardiac autonomic modulation in young individuals with T1DM. Further larger-scale confirmatory studies are warranted to confirm the potential risks of vigorous exercise in this population [45]. The latest guidelines published in 2018 by the International Society for Paediatric and Adolescent Diabetes (ISPAD) recommend that children (aged 5–11 y) and adolescents (aged 12–17 y) should aim for 60 min or more per day of MVPA physical activity and minimize sedentary time, and participate in vigorous intensity exercise, muscle and bone strengthening exercise at least three times a week [46]. In our study, individuals living with T1DM did not achieve the minimal time of daily MVPA (60 min). Moreover, total daily physical activity count and the time spent in MVPA were significantly lower in individuals with T1DM than apparently healthy peers without diabetes (mean difference − 36 min). Reduced and insufficient physical activity during childhood is an important risk factor for CVD [47]. Results of an observational study of children and adolescents (aged 4–18 years) demonstrated associations between increased time spent in sedentary activities with decreased levels of physical activity and related cardiovascular risk factors [48]. Furthermore, cardiorespiratory fitness was significantly reduced in individuals with T1DM compared with apparently healthy peers without diabetes in our study. Similarly, previous research also found that adolescents with T1DM have a lower aerobic exercise capacity when compared with normal controls [49, 50]. However, well-documented evidence of randomized controlled trials showed that there was improvement in cardiorespiratory fitness with exercise training [51–54]. These results highlight the importance to engage in regular physical exercise for persons living with T1DM. Importantly, very small volumes of physical activity appear to have significant health benefits [1, 31].

A positive association between HDL-C and METs, and a trend for a negative association between HDL-C and time spent in sedentary behaviour was found in the T1DM group. Furthermore, triglycerides were negatively associated with daily physical activity counts and METs, and positively associated with time spent in sedentary behaviour in youth with T1DM, while these relationships were not shown in the apparently healthy peers without diabetes. These results are in agreement with previous studies in youth with T1DM that regular physical activity was associated with improved metabolic control and lipid profile in adolescents with T1DM [42, 55]. However, age, sex, pubertal stage, BMI z-score, and insulin treatment may affect an individual’s physical activity variables and lipid profiles. Therefore, it is expected in multivariable analyses that potential confounding factors (age, sex, pubertal stage, BMI z-score, and insulin treatment) would decrease the association with physical activity variables and lipid profiles. Martin and colleagues revealed that each MET (approximately 3.5 mL·kg^−1^·min^−1^) increase in cardiorespiratory fitness was associated with a 25% point reduction in all-cause mortality [56]. Furthermore, in a systematic review and meta-analysis of physical activity and major chronic diseases showed that increased exercise, from achieving 11.25 MET-hours per week (675 MET-minutes per week), may be effective to decrease incidence and mortality of cardiovascular disease by 17% and 23%, respectively [57]. Therefore, our observation suggests that increased daily physical activity has positive effects on CVD risk factors. Interpretation of these findings should be considered with the confounding effects of age, sex, pubertal stage, BMI z-score, and insulin treatment. Further research in this field is warranted.

## Limitations

The major strengths of the study include a well characterized cohort of Chinese youth living with T1DM and apparently healthy peers without diabetes and the use of an objective and reliable method to assess the physical activity levels and aerobic fitness. There were some potential limitations with our study. First, because of small sample size, our study may have been underpowered to detect significant changes in some variables. Second, wGT3x-BT triaxial accelerometers are water-resistant, but not waterproof, participants were required to remove the device when they engaged in aquatic activities such as swimming, which would lead to a potential underestimation of total physical activity levels. We only measured physical activity for one week, and this may not reflect the annual physical activity pattern. Third, the cross-sectional nature of this study does not allow determining causality; however, there was compelling information demonstrating important relationships between regular physical activity and the risk for CVD in youth living with T1DM. A randomized controlled trial including a structured exercise training program would be required. Furthermore, there was a potential for selection bias since not all diabetic individuals who were approached agreed to participate in the study (48 out of 79 diabetic participants consented to participate the study). It is possible that the individuals who were more sedentary or inactive may have been less willing to participate in the study. Additionally, the sample size in apparently healthy group was less than T1DM group, and the power is based on the smaller sample.

## Conclusions

Our findings provide evidence that, despite their young age and short duration of diabetes, Chinese youth living with T1DM exhibit proatherogenic lipid profiles characterized by higher total cholesterol, LDL-C, and triglycerides, which is similar to that of other ethnicities. Furthermore, Chinese youth living with T1DM showed lower physical activity levels and VO_2_max compared to apparently healthy peers not living with diabetes. Being physically active may reduce the risk for CVD in youth living with T1DM when confounders of age, sex, pubertal stage, BMI z-score, and insulin treatment were included. As such, further larger-scale experimental design studies (e.g. randomized controlled trials) are warranted to confirm the findings. Accordingly, this study provides compelling evidence supporting the promotion of physical activity in youth living with T1DM to reduce the risk for cardiovascular morbidity and premature mortality.

## Data Availability

The datasets used and/or analysed during the current study are available from the corresponding author on reasonable request.

## Abbreviations

T1DM: Type 1 diabetes mellitus
CVD: Cardiovascular disease
LDL-C: Low-density lipoprotein cholesterol
HDL-C: High- density lipoprotein cholesterol
VO_2_max: Lower maximal oxygen power
METs: Metabolic equivalents
MVPA: Moderate to vigorous intensity physical activity
VO_2_R: Oxygen uptake reserve
BMI: Body mass index
WHO: World Health Organization
DXA: Dual energy X-ray absorptiometry
SD: Standard deviation
CPM: Counts-per-minute
MDI: Multiple daily injection
CSII: Continuous subcutaneous insulin infusion
CGM: Continuous Glucose Monitoring
SMBG: Self-monitoring of blood glucose
HbA1c: Glycosylated hemoglobin
CI: Confidence interval
ISPAD: International Society for Paediatric and Adolescent Diabetes
